# Dazzle camouflage: benefits and problems revealed

**DOI:** 10.1098/rsos.240624

**Published:** 2024-12-04

**Authors:** P. George Lovell, Rebecca J. Sharman, Tim S. Meese

**Affiliations:** ^1^ Division of Psychology & Forensic Sciences, Abertay University, Bell Street, Dundee DD1 1HG, UK; ^2^ Centre for Vision & Hearing Research, Aston University, Aston Street, Birmingham B4 7ET, UK

**Keywords:** camouflage, dazzle camouflage, vision, perception, direction, psychophysics

## Abstract

During WWI, ships were painted in high-contrast 'dazzle' patterns believed to distort, among other things, submariners' perceptions of direction when aiming their torpedoes, but was this strategy effective? Here, we investigated the effects of different camouflage patterns, including versions used in the war, on the perceived direction of travel for a three-dimensional computer model of the RMS Mauretania. The results of this study showed that texture gradients ‘twisted’ the perceived direction of the ship, the effect being ~10° for a regular pattern of circles. We also found a second, larger effect, 'hysteresis', that biased perceived target directions to parallel the horizon for directions of travel within approximately ±30° of 90° (left–right). Hysteresis persisted outside this central plateau, causing perceived directions to be offset from veridical. The twist and hysteresis effects combined linearly and were constructive (enhancing protection) or destructive (diminishing protection) depending on the directions of (i) travel and (ii) the 'twisting' texture gradients. However, the strength of hysteresis reduced as a function of experience. Our simulated torpedo attacks suggest that systematic perceptual distortion of direction by dazzle might have been effective only where submariners had low hysteresis and ships were fast enough to benefit from the perceptual error imposed by twist.

## 1. Introduction

In the early twentieth century, when a submarine launched a torpedo attack on a ship, accurate aiming was essentially a question of geometry. In WWI, submariners faced competing demands in deciding how to position themselves. Being close to the target improved the chance of a hit but put the submarine in danger of detection, whereas attacking from afar was safer but incurred the cost of increasing the risk of a miss. In general, submariners attacked between these extremes, aiming ahead of the target to allow for the travel time of the torpedo. This strategy required an estimate of the target’s future position derived from estimates of several factors including direction of travel and speed (refer to electronic supplementary materials, S1 and S2, for some details on how this was done). Marked errors in the estimates of these parameters could lead to failure unless the ship was very large, close or slow. Nonetheless, during WWI, submariners developed considerable skill in this task.

**Figure 1.**
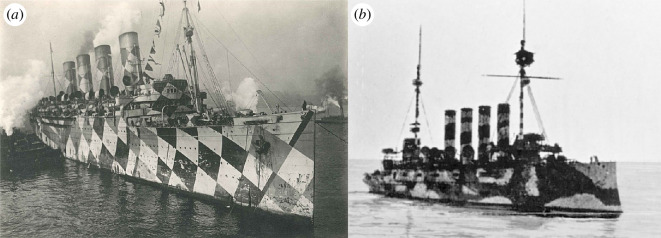
(*a*) RMS Mauretania with camouflage designed by Norman Wilkinson. (*b*) HMS Argonaut with camouflage designed according to Graham Kerr’s specifications. Mauretania image, public domain, New-York Tribune, 8 December 1918. Argonaut image, with permission of University of Glasgow Archives & Special Collections, DC006/624.

On the British side, it became imperative to find ways of making the enemy’s task more difficult. In 1917, a form of camouflage that become known as dazzle^1^ was applied to thousands of merchantmen, troop carriers, escorts (destroyers and cruisers) and other ships (for reviews [1–7]). To whom the credit for this development should be attributed is surrounded by controversy—the biologist Graham Kerr and the artist Norman Wilkinson both made claims (for details [1,3] and electronic supplementary material, S2, all of which favour Kerr). Dazzle designs were different for each ship but always consisted of high-contrast patterns, often achromatic. Photographs of example designs are shown in figure 1 for RMS Mauretania (a Wilkinson design) which was used as a troop ship and the cruiser, HMS Argonaut (a Kerr design). It was Wilkinson’s work that won favour with the British Admiralty but a detailed account of what Wilkinson was trying to achieve with any single design appears not to exist [2]. Nonetheless, there is no doubt that the primary aim of the dazzle approach was to distort the perceived form of the ship, thereby (i) interfering with the identification of class and degrading estimates of speed and size and (ii) distorting the perceived direction of travel.

If successful, the approach would impose targeting errors on submariners, thereby saving shipping, cargo and lives. Unfortunately, it is difficult to determine whether this audacious approach achieved its aims. The number of allied ships hit (sunk, damaged or captured)^2^ in the year dazzle was introduced (1917) was substantially higher (3725) than in the previous year (1523) but this was presumably due to the threefold increase in U-boat activity during that period. Losses declined again in 1918 (1649) but this is traditionally attributed to the introduction of the convoy and escort system in mid-1917. Not surprisingly, controlled experiments to compare the success of dazzle ships against untreated ships of similar types and sizes and under similar settings were not conducted under battle conditions during the war. Shortly after, Blodgett [8] reported some elaborate experimental work using small-scale model ships but did not conduct a systematic investigation of ship directions relative to the periscope.

More recent work offers some support for the value of dazzle, where misperceptions of speed have been reported for fast-moving and high-contrast zigzag and checkerboard patterns [9]. On the other hand, in a touch-screen task, dazzle targets were picked out more quickly than their plain counterparts (for a review [10–12]). However, absolute target speed is a critical factor here. In these two experiments, the key speeds were 20°/s [9] and 26.7°/s [12]. In contrast, a 100 m ship travelling at its maximum hull speed of 45.28 kph would move at 0.7°/s when viewed side-on from a distance of 1 km. For these conditions, it would take 28 s for the ship to travel across the 20° field of vision of a German targeting periscope. Therefore, for large ships at large distances, the direct perception of speed (as opposed to that which might be assumed—refer electronic supplementary material, S1) was probably irrelevant because the visible motion would be so slow. Distortions in the perception of direction, on the other hand, are another matter.

Here, over a hundred years after the Royal Commission on Awards to Inventors (TNA. TS32/19B) reviewed the issue (refer electronic supplementary material, S3), we provide a long overdue experimental investigation into the effects of dazzle camouflage. To do this, we measured and compared the perceived direction of travel for simulated ships on the open sea. Our ships had either a neutral grey design (which was common around the outbreak of war [2]), or one of five different camouflage patterns. Those developed by Wilkinson & Kerr were numerous, though several were quite similar [3]. The patterns for RMS Mauretania (Wilkinson) and HMS Argonaut (Kerr) (figure 1) were chosen for the current study because usable images were readily available and, from casual inspection, they looked rather different. For example, the Wilkinson design involves well-defined geometric shapes and contours, whereas the Kerr design is formed from light and dark blotches.

Another important factor when designing our stimuli was the concept of texture gradients [13]. These are the smooth changes in the retinal image of element size, shape and density that derive from perspective projection when viewing a textured planar surface at a slant [14]. For example, consider a surface in the fronto-parallel plane covered in an even texture of circular spots and then slanted away from the onlooker so that the right-hand vertical edge is further away than the left-hand one (for a photograph of this arrangement; refer electronic supplementary material, S4). In the retinal image, the spots on the right will be smaller than the spots on the left, with a smooth gradient of spot size, shape and density between them. Conversely, we can return to a plain surface in the fronto-parallel plane and render it with a photograph of the texture gradient from the previous slanted arrangement. Experiments with related stimuli show that the surface is now perceived as slanted, consistent with the earlier manipulation, even though the physical surface remains fronto-parallel [14–16]. Since some dazzle designs (including that on Mauretania) suggest forms of texture gradients to us (sometimes called forced perspective in this context), we reasoned that these might lead to systematic misperceptions of the direction of travel. To test this further, we also devised our own dazzle patterns based directly on this principle.

## 2. Material and methods

### 2.1. Participants

Sixteen participants with normal or corrected-to-normal vision took part in the study and gave informed consent. Three participants were Abertay University psychology students and received credit from the co-operative participant pool for taking part. Four participants were recruited through general social media, and a further nine participants (including the author, P.G.L.) were members of a Facebook group created for those interested in sailing (Pocket Yachts and Trailer Sailors). To comply with General Data Protection Regulations (GDPR) no demographic information was retained apart from the number of years of experience in sailing which we predicted would relate to accuracy in the task. All procedures were approved by the Abertay University Ethics Board and were in accordance with the Helsinki Declaration.

### 2.2. Stimulus generation

#### 2.2.1. Camouflage patterns

**Figure 2.**
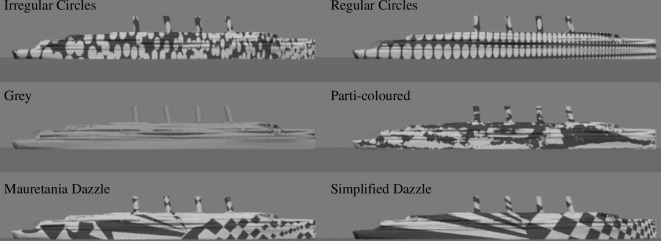
The six camouflage conditions used in the psychophysical experiment (five patterns and neutral grey). These static ships point to the right/east, implying a direction of travel of 90°.

The six camouflage conditions used in the experiment are shown in figure 2. They consisted of a neutral grey condition and five dazzle-like patterns. The patterns from HMS Argonaut (parti-coloured) and RMS Mauretania (Mauretania dazzle) were digitally traced from original photographs [3] using Adobe Photoshop, v. CS6. A modified version of the Mauretania scheme (simplified dazzle) was adapted by hand to simplify the features on the left and right sides of the image and to emphasize the texture gradients. The regular circles pattern (23 pixels in diameter, 29 pixels between centres) was created by placing circles on a grid with equal *x*- and *y*-spacing (*n* = 57 elements along *x*). The irregular circles pattern was created by positioning circles randomly, with the sole constraint that they should not overlap, though they could touch, until the quantity of circles matched that of the regular circles pattern.^3^ To generate the texture gradients for the two circle patterns, the images were displayed on a flat LCD computer screen (Hanns-G 28inch; HANNspree, Taipei, Taiwan), rotated to an (arbitrary) angle of 47° and photographed using a digital camera (DMC-LX7; Panasonic, Kadoma, Japan) placed 192 mm from the centre of the image. This general approach is not unlike that used by Warner in a forced perspective type of design in the United States [4]. The diameters of the elements projected onto the model varied from 5 to 11 m at the scale of the model. The full set of two-dimensional camouflage images used in the rendering are shown in electronic supplementary material, S4. The camouflage patterns were mirrored on the two sides of the ship, so that the bow looked the same regardless of which side was viewed.

We chose to use achromatic images for simplicity and consistency. Many of Wilkinson’s schemes were painted in black and white, and there are no colour photographs of ships from this time, so knowing the true colouring is difficult in some cases (e.g. Kerr’s Argonaut). The true colours for Mauretania are known from Admiralty records and were, essentially, monochromatic shades of blue-grey, not the psychedelic shades that a well-known publicity painting showed her in [17].

Based on an informal visual inspection, four of our stimuli contained evidence for texture gradients: the two Mauretania conditions and the two circle conditions. We did not counterbalance the direction of these gradients (stern-to-bow versus bow-to-stern) for three reasons. (i) Imposing a misperceived swing away from the observer was predicted to have a greater effect than a swing towards, consistent with the views of Lieutenant Loyd A. Jones from Eastman Kodak Company, who was heavily involved in the US dazzle camouflage project [2]. (ii) In the published examples of schemes where texture gradients are evident, this is the direction of application, particularly those from the U.S. Watson/Norfolk system [2]. (iii) By way of maintaining participant engagement with the task, we did not want to increase the number of trials unduly. Note that because we presented ships with directions around the compass (refer §2.3), there were as many trials with texture gradients to the left as to the right.

#### 2.2.2. Digitized model ships

For consistency, all camouflages were rendered onto the same virtual model which was a three-dimensional scan of a physical model of RMS Mauretania (Editions Atlas, S. A., China) using a three-dimensional scanner (3D3 scanner with Flexscan software; Mech Innovation Limited, Warwickshire). The field of view of the ‘camera’ in the environment was adjusted to produce a ship size consistent with a viewing distance of 750 m under real-world viewing conditions and with a periscope height of 1 m. Further technical details about the scanning, rendering of camouflage, grey levels and simulated illumination can be found in electronic supplementary material, S5.

### 2.3. Procedure

The experimental software was written using PsychoPy Builder, v. 1.90.3 [18] which automatically translated the experiment into JavaScript. The experiment was run online.

To ensure the ships had a consistent retinal image size across participants, we scaled the stimuli according to local screen dimensions and each participant’s preferred viewing distance. This was achieved by following the technique described by Li *et al.* [19] (refer also electronic supplementary material, S5).

**Figure 3.**
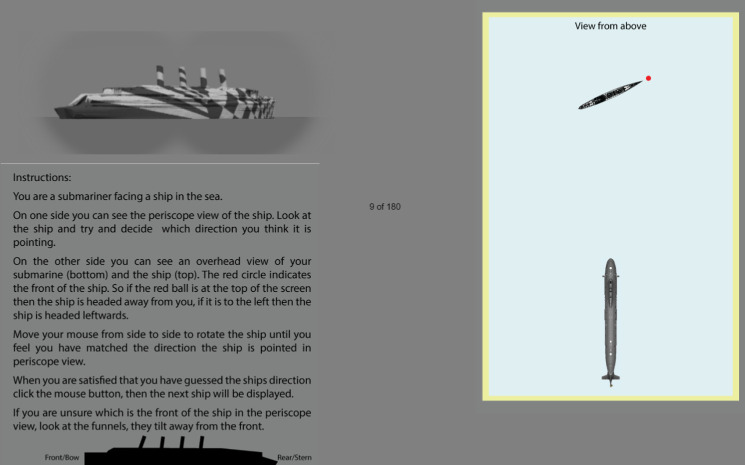
Screen capture of the experiment in progress. Participants viewed the stimulus ship through a binocular-like window (top left). An overhead ‘chart’ (right) featured a submarine icon at the bottom always facing upwards (north/0°) with the response ship placed above that. Participants rotated the response ship on the right until it matched the perceived direction of travel for the stimulus ship on the left. In this example, the stimulus ship carries the simple-dazzle colour scheme and has a compass heading of 40° (NE).

The experimental display is shown in figure 3. The image of the stimulus ship was static and the instruction panel remained *in situ* for the entire experiment. The other side of the display contained icons of a 'response ship' for which the initial direction was randomized between 0° and 359° (0° being north/upwards in the display) and a static submarine. When participants moved their mouse (or finger in the case of a trackpad) from side to side, the target icon rotated in real time (i.e. the mapping between icon direction and the mouse/trackpad depended only on the *x*-coordinate). Participants were asked to match the direction of this icon to that of the stimulus ship. The red dot is explained in the instruction panel in figure 3 and was included to reduce the potential for misidentifying the bow and stern of the icon. Participants recorded their responses with a mouse click, which also initiated the next trial. No response feedback was given. The assignment of the content to left and right panels of the display (figure 3) was randomized across participants, but consistent within participants.

On each trial, and for each camouflage condition, the direction of the stimulus ship was randomly selected without replacement, from between 0° and 350° in steps of 10°, excluding directions where the ship was facing largely towards or away from the observer (350°, 0°, 10°, 170°, 180° and 190°). These directions were omitted because (i) at these angles, both sides of the ship were visible (and severely foreshortened) and we wanted to restrict the analysis to the single sides of the original two-dimensional patterns (figure 2) and (ii) because WWI attacks on the bow or stern would have been infrequent due to the small visual angle of the target. The 280° condition was omitted for 13 of the 16 participants because of a transcription error in the coding of the experiment. Each pairing of direction and pattern was presented once, giving 174 unique trials for each participant. After the final trial, participants were debriefed, thanked for their co-operation and advised to close the experiment’s browser window.

## 3. Results and discussion

### 3.1. Human results: misperceptions of ship directions

Trials were excluded from further analysis where the absolute difference between the response and the true direction of the ship was greater than 90°. Out of a total of 2802 trials, 93 were rejected (3.3%). In these trials, the error was typically around 180°, suggesting that the bow and stern ends of the ship had been misidentified. A *χ*^2^ analysis of the number of excluded trials for each pattern revealed no significant variation in the prevalence of these trials for each pattern type.

**Figure 4.**
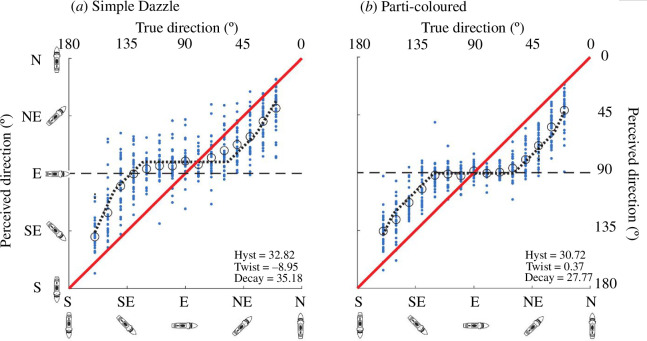
Raw data (small blue circles), average results (large black circles) and model fits (black dotted curves) for two example conditions in a two-dimensional direction space for the target ship (perceived versus true). (*a*) Simple-dazzle camouflage. (*b*) Parti-coloured camouflage. On both axes, 0° and 180° denote ships pointing away from and towards the north-facing observer, respectively (both axes run from south to north for the direction of the stimulus ship). The red line is the contour of veridicality. Data points that fall above the line of veridicality indicate that the bow of the ship appeared north of the true direction, those below the line indicate a perceived direction south of veridical. Parameter values for hysteresis, twist and decay (lower right in each plot) are the means of the three-parameter fits (not shown) to individual results (small blue circles) in degrees.

The camouflage patterns were the same on both sides of the stimulus ships allowing results to be collapsed across mirrored directions (e.g. NW with NE, W with E and SW with SE). Figure 4 shows the results for two example conditions (simple dazzle and parti-coloured) where perceived direction (determined by the setting of the icon in figure 3, right) is plotted as a function of the true reference ship direction (exemplified by the image rendering in figure 3, left). Note that the numerical inversions of the compass axes in figure 4 are to aid intuition so that northerly twists of direction away from the observer relate to intuitive upward shifts in the graphical space. The results for all six conditions (electronic supplementary material, S6) had the same characteristic serpentine form with two main features: (i) a central horizontal section; and (ii) a pair of diagonal sections that run largely parallel with the contour of veridicality (the red diagonal line through the origin in figure 4). We refer to the central horizontal section (and its impact on the rest of the function) as hysteresis because the perceptual effect is a 'clinging' to the horizontal (90°) over a range of directions until this pull withers and eventually decays. Alternatively, this effect could be described as 'horizon bias'. In figure 4*b* this feature of the results means that (i) for directions between 60° and 120°, the perceived direction of the stimulus ship was parallel to the horizon (90° on the *y*-axis); and (ii) the distance of the diagonal sections from veridical depends on the magnitude of hysteresis.

**Figure 5.**
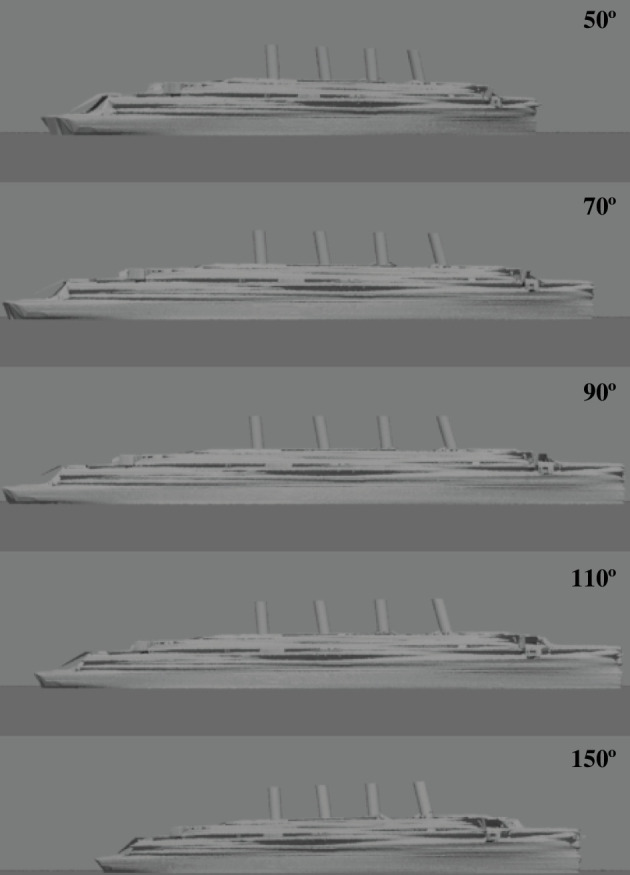
Five examples of direction of travel (refer insets) for the neutral grey camouflage condition. Only the ship at 90° is parallel with the horizon.

We can get a sense of the perceptual error from hysteresis in figure 5 which shows images of the neutral grey ship pointing in the true directions shown by the insets. The middle three ships all tend to look parallel with the horizon, though the ships at 70° and 110° subtend a smaller visual angle and are perceptibly shorter than the one at 90°.

Another effect noticeable in some of our data was the tendency for the whole curve to be shifted north relative to veridical. We refer to this effect as ‘twist’ and it can be experienced perceptually in figure 2. These ships are all parallel to the horizon (direction of 90°) but in the top right image (regular circles), for example, the bow appears twisted away from us, consistent with the texture gradient in the camouflage (refer §1). This effect not only applies across much of the direction range tested but also combines perceptually with hysteresis. This manifests in the results (figure 4*a*) in two ways. First, the horizontal segment of the hysteresis effect is above 90° (by about −10°), indicating a northern twist away from parallel with the horizon. Second, the data region in the lower left of the plot is more distant from veridicality than the data region in the upper right.

**Figure 6.**
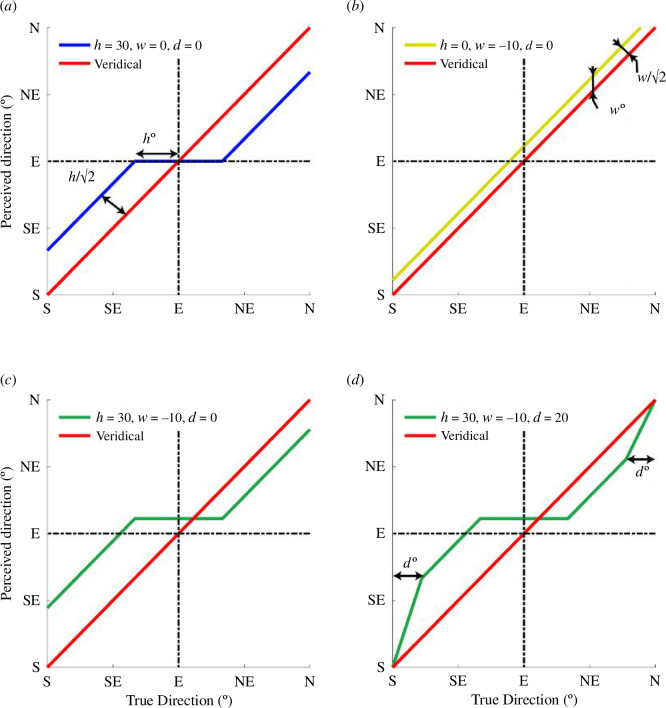
Example behaviours of our three-parameter descriptive model in a two-dimensional direction space for the response ship (perceived versus true). (*a*) *h* (hysteresis) = 30°. (*b*) *w* (twist) = −10°. (*c*) The linear combination of *h* = 30° and *w* = −10°. (*d*) The same as (*c*) but with the addition of *d* (decay) = 20°. Note the similar form of (*d*) and the results in figure 4*a*.

The theoretical form and interaction between twist and hysteresis is shown in figure 6, where we develop a general three-parameter descriptive model of ship-direction perception. The figure illustrates hysteresis alone of 30° (figure 6*a*), twist alone of −10° (figure 6*b*), a linear combination of the two (figure 6*c*) and a combination of the two with the addition of a decay parameter (figure 6*d*) justified and formalized in §3.2. In our illustration, the different sized effects (of *h* and *w*) combine constructively and destructively in the lower left and upper right regions, respectively (figure 6*c*). This illustration is similar to the main features of the human results for regular circles (figure 4*a*). Note that while we suppose the two effects have quite different origins, they are expressed in directly comparable units. Hysteresis is given by the horizontal distance *h*, while twist is given by the vertical distance *w*; both relate to perpendicular offsets of the effect curve from veridicality by a factor of √2 (figure 6*a*,*b*). In other words, if *h*=|*w*|, then the shifts from veridicality are of the same magnitude for each effect. This mix of constructive and destructive interactions has consequences for the benefit of dazzle, as we will see in §3.5.

### 3.2. Parameter estimation for the human results: descriptive modelling

To estimate the values of twist, hysteresis and decay for each camouflage condition we fitted the results with our three-parameter descriptive model (*h*, *w* and *d*; refer figure 6) using an exhaustive search technique to minimize the RMS error of the fit (black dotted curves in figure 4). The inclusion of decay (*d*) was motivated by two factors. First, and most importantly, we reasoned that for our symmetrically camouflaged ships, perceived direction would return to veridical for ships facing directly towards or away from the observer (0° and 180°). Second, although we did not gather data for directions around 0° or 180°, most of our six camouflage conditions displayed empirical evidence for the onset of this effect (e.g. refer to the left-most points in figure 4*a* and electronic supplementary material, S6, for the other conditions).

Formally, the value of *d* is the distance (degrees) from the left and right ends of the *x*-axis where the model curve, carrying the effects of *h* and *w*, turns back towards veridical by way of a straight line (refer figure 6*d*). Thus, when all parameters (*h*, *w* and *d*) are greater than zero, our three-parameter model has five straight line segments (figure 6*d*); the three in figure 6*c*, plus the decay sections at each end. The value of *d* has no theoretical importance in this study but including it in the fits cleared up systematic residuals and thereby improved the quality of our numerical estimates of *h* and *w*. Statistical analyses (refer the MANCOVA in the next section) showed that *d* did not co-vary with camouflage condition or sailing experience, and we do not discuss its meaning further.

**Figure 7.**
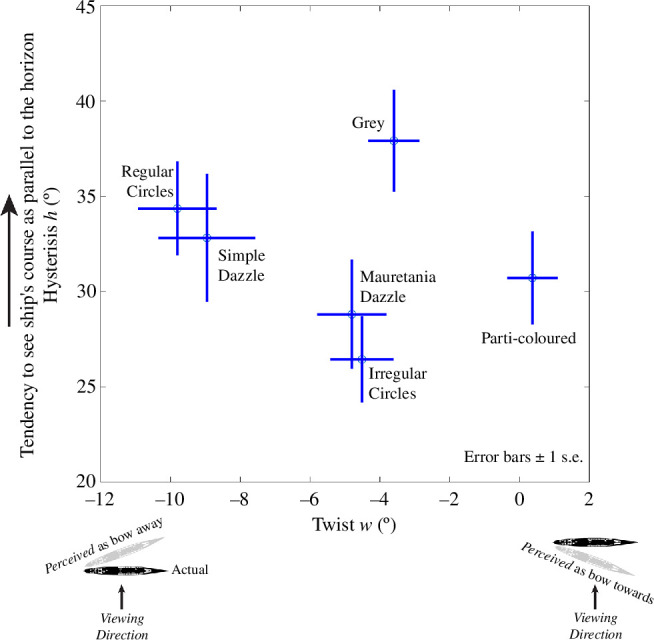
Values of hysteresis (*h*) and twist (*w*) for each camouflage condition from fitting a three-parameter descriptive model to the human results (refer figure 6 for parameter definitions). The data points are means across the 16 fits to individual observers and error bars are ±1 s.e. Note the different ranges on the two axes for directly comparable units (directions in degrees). The icons at each end of the *x*-axis visualize the twist effects.

The bi-variate plot of figure 7 shows values for the two parameters of interest (*h* and *w*) derived from the descriptive model fitting for each camouflage condition. There is no obvious clustering of the results in this space (we will come to the proximity of regular circles and simple dazzle in §3.4.2), nor any obvious relation between the two parameters across stimulus conditions. Nonetheless, there are three striking observations. First, hysteresis was strongly evident in all conditions and substantially greater in magnitude than the twist effect, at least a factor of three in all conditions. Second, the hysteresis effect for the neutral grey condition (figure 5) was larger than for any of the five camouflage patterns. Third, four of the camouflage patterns (both types of circles and both types of Mauretania dazzle) produced a notable twist effect in the range of −4° to −10°. Note that the negative signs of *w* indicate that the misperceptions involved a swinging of the bow away from the observer (refer figure 6) consistent with the direction of the texture gradients in our stimuli. On this matter, we also note that Kerr’s parti-colour scheme (figure 2) invoked no significant twist, and the neutral grey condition produced a twist effect only slightly shy of those for two of the pattern conditions that we had supposed included texture gradients (irregular circles and Mauretania dazzle).

### 3.3. Statistical analysis of model fitting to the human results

To examine the effect of our participants’ sailing experience (measured in years), and to provide statistical enquiry into some of our observations above, we conducted a one-way MANCOVA with length of experience as a covariate. The values of *h*, *w* and *d* were dependent variables, and the stimulus (camouflage) condition was the independent variable. There was a main effect of pattern type (*F*(15,267) = 5.05, *p* < 0.001), and sailing experience was a significant covariate (*F*(3,87) = 4.24, *p* = 0.008). Bonferroni corrected post hoc tests demonstrated that there were significant differences in hysteresis fits for grey versus Mauretania dazzle (*p* = 0.03, Cohen’s *d* = 0.94); and grey versus irregular circles (*p* = 0.03, Cohen’s *d* = 0.95). All twist fits were significantly different (with large Cohen’s *d* effect sizes of between 0.94 and 2.02) except grey versus Mauretania dazzle; grey versus irregular circles; regular circles versus simplified dazzle and Mauretania dazzle versus irregular circles (full details of the post hoc tests can be found in electronic supplementary material, S7, and the OSF Repository).

Sailing experience also influenced hysteresis (*p* < 0.001), but not twist (*p* = 0.899) or decay (*p* = 0.218). However, we did not ask our participants their age so we cannot rule out the possibility that the effect of sailing experience was in fact a more general one of visuo-cognitive experience through age. For simplicity, we refer to this factor as 'sailing experience' in what follows, as this is the factor we recorded, but caution that this might plausibly be correlated with a different causal factor such as visuo-cognitive experience with age. The effect of hysteresis was negatively correlated with sailing experience (i.e. hysteresis reduced with years of experience), but whether this reflects differences in motivation or perception remains unclear. Put another way, we cannot know whether subtle visual cues were more readily available to experienced participants compared to others, or whether they simply put more effort into giving precise responses.

### 3.4. General discussion of the human results

#### 3.4.1. Hysteresis (or horizon bias)

Our hysteresis/horizon effect (vertical position in figure 7) is a form of perceptual bias where the perceived direction of a distant ship parallels the horizon. In our experiment, participants were required to identify the direction that stimulus ships travelled which first required the identification of bow and stern. Preliminary work showed that some naive participants were occasionally confused by this, even for the neutral grey ship. We reasoned that this kind of categorical error would degrade the quality of our results but was unlikely to be one made by experienced submariners (for our camouflage designs, at least. Refer §3.5.3, §4.1 and §4.2 for discussion). This is why we used a red dot to mark the bow within the map view and drew attention to the slope of the funnels in the instructions (figure 3). With bow and stern identified, the general direction of rightward (eastward) or leftward (westward) travel is determined. Without evidence to the contrary, a reasonable default that minimizes overall error is to select the direction that is midway between the range of possibilities, 90° (east) or 270° (west), respectively. Thus, the default response is a direction parallel with the horizon. As we discuss below, this default is presumably subject to further additive biases, but first, we consider the perceptual evidence that overcomes the default.

The neutral grey camouflage ship serves as a baseline against which the various pattern treatments can be compared. The grey condition had a greater hysteresis than any of the camouflage patterns, extending across much of the direction range shown in figure 5. This means that the pictorial cues for depth in these images (top and bottom of figure 5)—e.g. the three-dimensional relief at the stern and bridge of the ship and the heights of the smokestacks—was doing little to overcome our participants' horizontal default. We suppose that experienced submariners would not overlook these cues, consistent with our finding that hysteresis is negatively correlated with experience in sailing. Presumably, for directions outside 90° ± 40°, even our less experienced participants were able to pick up on the pictorial shape from shading cues and other cues (e.g. length; refer figure 5), though underestimated their meaning owing to the pull of hysteresis.

The five pattern conditions each produced lower values of hysteresis than the grey condition. This suggests that the luminance contours of the patterns facilitated our participants’ ability to pick up on the cues for three-dimensional relief [20], for Mauretania dazzle and irregular circles at least, where the differences were statistically significant (electronic supplementary material, S7). However, it is unclear why hysteresis varied across camouflage patterns, in particular why hysteresis should be smaller for irregular circles compared to regular circles (a difference of around 8°; refer figure 7), though this was shy of significance when Bonferroni corrected (electronic supplementary material, S7). It might be that the serendipitous fall of the patterns on critical regions (such as the bridge) was important, enhancing or masking the benefits of the texture for the observer, rather than the properties of the patterns in general.

#### 3.4.2. Effect of pattern: twist and perceptual bias

The horizontal positions of data points in figure 7 show the magnitude and sign of twist for each condition. Figure 2 suggests that regular circles and simple dazzle carry the strongest impression of texture gradients, consistent with these being the conditions that produced the greatest twist (around −10° and −9°). This is markedly greater than that found with no patterning (neutral grey = −3.6°) (refer figure 7), providing good evidence that dazzle-type patterns can impose a bias on a ship’s perceived direction of travel. However, the twist effects for Mauretania dazzle and irregular circles were barely greater than for the grey condition, indicating that dazzle treatment in general does not guarantee a twist effect. Indeed, there is no reason to expect that it would because twist is a manifestation of perceptual bias and that requires some form of systematic bias in the camouflage design that is meaningful to human observers.

The only difference between the regular and irregular circles conditions is the positioning of the circles, yet this had a marked effect on our estimate of twist, decreasing it by about 5°. Texture gradients contain three cues to slant [14], and these are not changed by randomizing the locations of the elements, but human perception of slant from texture falls short of the ideal observer [21]. Instead, there is a visual preference for so-called perspective convergence, which is signalled by reduced contrast energy in the direction of slant [22,23]. On this line of thinking, because texture irregularities break the spectral structure, the perceived slants and discrimination sensitivities for irregular surfaces tend to be less than for regular grid patterns due to the reduction in linear cues [23]. Therefore, randomly positioning the circles could have introduced more uncertainty into the condition reducing both forms of bias. A further factor is that the randomization process for positioning our irregular circles allowed them to touch but not overlap. This might have degraded the perceptual quality of the texture gradient, further explaining why irregular circles produced less twist than their regular counterpart in our experiment.

Our simplification of Mauretania’s dazzle scheme increased the twist effect by over 4° (refer the two ‘Mauretania dazzle’ and ‘Simple dazzle’ data points in figure 7). We attribute this to the emphasis that our modification made to the texture gradient in the camouflage: the clearer the texture gradient (presumably achieved by our modification), the greater the impact on twist [23,24].

In sum, for the two arrangements where we synthesized stimuli to enhance texture gradients (regular circles and simplified dazzle), the value of twist was markedly greater than for their counterparts.

#### 3.4.3. Twist effects for the neutral ship

The marked twist effect for the neutral grey condition was unexpected. It is a challenge to construct an argument in terms of response bias because the starting direction of the response ship was randomized for each trial and the results are collapsed over mirror directions for eastward and westward (left and right) facing ships. Could there be a perceptual origin for the bias in the neutral camouflage? Close visual inspection of figure 2 reveals a hint of a slight turn of the stern towards the observer, presumably owing to the shading, and perhaps this imposes a perceptual turn of the bow in the opposite direction. It is possible that this unintended consequence of the rendering underlies the small twist effect for our neutral condition.

### 3.5. The impact of twist and hysteresis on torpedo strikes: simulation modelling

We have shown that the perceptual error in judging the direction of a ship depends on the camouflage the ship carries. However, this does not tell us what impact this error has on the successful aiming of a torpedo. Indeed, two of our findings raise serious issues for the supposed benefit of adding dazzle treatment to a neutral grey ship: (i) dazzle-type camouflage tends to decrease the detriment of the hysteresis effect (figure 7); and (ii) the addition of a dazzle-induced twist on a backdrop of hysteresis can both increase and decrease the magnitude of perceptual errors on different parts of the function (figure 6). To understand the impact of these factors we performed torpedo aiming simulations to calculate the hit rate under various combat conditions and camouflage treatments, characterized by our estimates of *h*, *w* and *d*.

#### 3.5.1. Simulation methods and their real-world background

Torpedoes are relatively slow-moving weapons. The WWI torpedo, G/6D, had a speed of 35 knots (64.8 kph) and a range of 3.5 km [25]. Therefore, as mentioned in §1, to strike a distant moving ship, submariners had to calculate the location at which the ship and torpedo would intersect and this required estimates of direction of travel, range and speed. WWI torpedo attacks often operated in multiple stages, for example, submerging, tracking the ship along a parallel path, taking positions perpendicular to the ship’s course and waiting until the ship was optimally placed before firing [26]. Here, we simulated only the final targeting decision, assuming the submarine was already at a suitable location.

A ship’s speed is limited by its hull speed, an interaction between the ship’s length and the bow wave it creates. As a ship approaches its hull speed, the bow wave grows and prevents further acceleration. The size of the bow wave varies as a function of ship length, shorter ships having a lower maximum speed. The ship speeds in our simulated attacks were based on 75% of their hull speeds which provides a fair match to reports for WWII era merchantmen and passenger ships which were often converted for other uses [27].

In each simulation, the true course of the ship was generated using pre-selected speed, range and direction parameters (see below). A perceived course was also calculated according to predicted perceptual errors as follows. We used various estimates of *h*, *w* and *d*, including those from our experiments, to determine the perceived direction of the ship. Speed estimates were based on the true values with additive variability drawn from a normal distribution with an arbitrary standard deviation (σ) of 10% (e.g. if the ship was travelling at 20 kts, then simulated estimates of perceived speed across attacks had a mean of 20 kts and a standard deviation of 2 kts). Errors in range estimation do not affect torpedo aiming accuracy (refer electronic supplementary material, S2) so these remained at veridical (arbitrarily), and no variability was added. We assumed that the G/6D torpedo speed was constant at 35 kts and had a range of 3.5 km. The optimal firing solution was calculated numerically for the perceived course and compared to the veridical location of the ship. The target ship was defined by an ellipse matched in size to the ship’s length (its width was 0.11 × length). For a ‘hit’ to be recorded the torpedo had to enter the ship’s ellipse during the simulation. Calculations were conducted in 1/20th s time intervals until the torpedo reached its maximum range.

Simulations were run for ship lengths of 50–300 m (hull speeds of 17–42 knots; simulated ship speeds of 13–32 knots)^4^ in steps of 50 m, at distances of 500, 750 and 1000 m, for directions of 0–360° in steps of 2.5°, and for each camouflage treatment in our experiment. We also ran a condition with identical parameters to those from the regular circles condition but reversed the direction of the twist (to +9.8°). Each ship length × range × direction × camouflage was run 100 times.

#### 3.5.2. Simulation results and discussion

**Figure 8.**
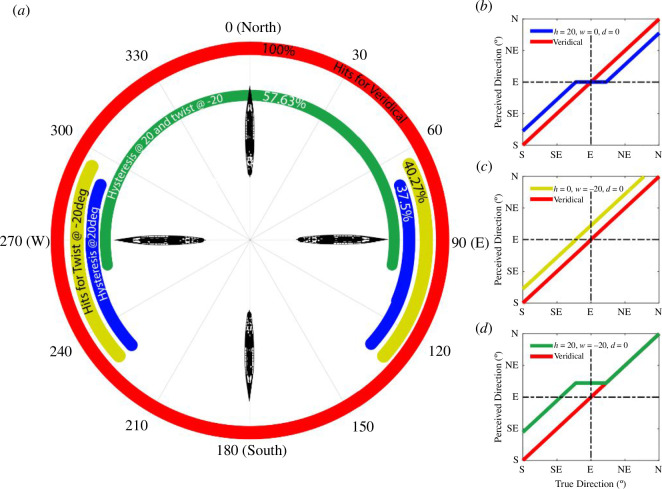
Results from simulations for a ship at a distance of 750 m, ship length of 150 m and speed of 22.5 knots. In these simulations, the decay (*d*) was 0° and there was no stochastic variability. The polar plot (*a*) shows the distribution of ship directions that result in successful torpedo attacks. The plots to the right show true direction versus perceived direction for exemplar model observers. The different colours represent hysteresis (*h*) of 20° (blue curve and (*b*)), twist (*t*) of −20° (yellow curve and (*c*)) and the sum of these two effects (green curve and (*d*)). The red curve is the veridical situation where misperception does not occur.

Figure 8 is an illustrative visualization of the simulations with the speed variability excluded. This shows the basic effects of twist and hysteresis around the compass using example values of *h* = 20° (blue) and *w* = −20° (yellow). Here, the effects are of the same magnitude and provide similar protection from submarine attacks (blue and yellow curves in figure 8*a*). This protection is weaker for directions around east and west, where the observational direction of the submarine is perpendicular to the direction of the ship. The protective effect extends further north than south because the distance to potential impact is greater when the ship is travelling away from the submarine, magnifying the consequences of errors in perceived direction. However, when the effects are combined (green curve) the proportion of hits increases considerably. In other words, the protection of hysteresis is diminished by camouflage patterns that create a perception of twist. This is because when twist and hysteresis combine, protection is abolished for more northerly directions owing to the region of ‘destructive interference’ where the two effects cancel out (figure 8*d*).

**Figure 9.**
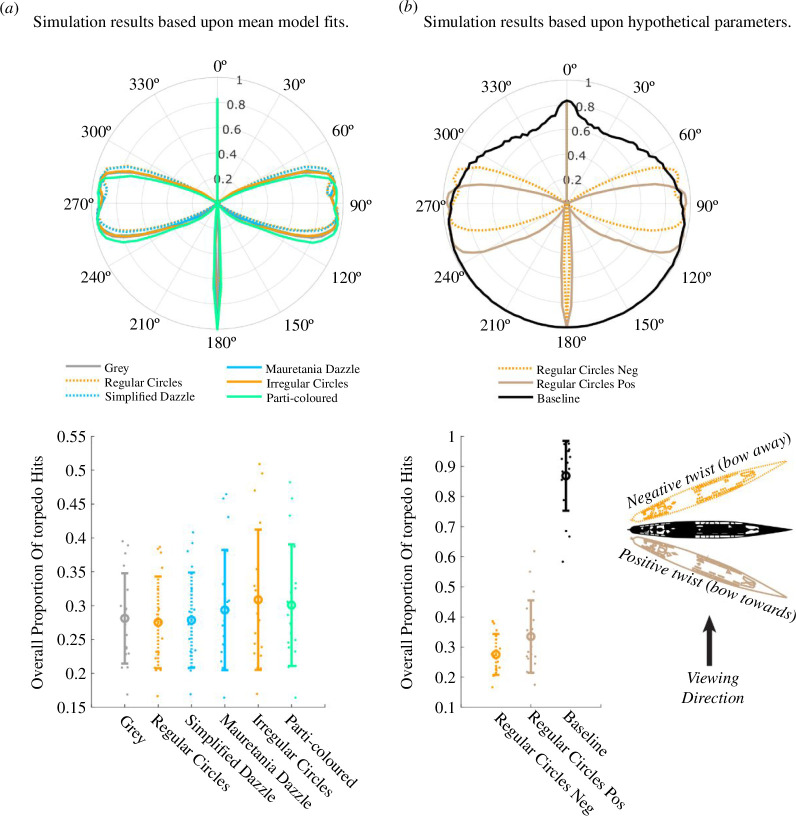
*Top*: Polar plots of simulated attacks for each camouflage type in the experiment (*a*) and for restricted set of analytic conditions (*b*). In all cases, the estimates of speed were varied according to a Gaussian distribution of errors with a standard deviation of 10%. Polar angle represents the direction of travel, and the distance from the origin represents the proportion of hits. Results are collapsed across ship length and range. *Bottom*: Proportion of successful hits across all directions, distances and ship lengths. The error bars show standard deviations of values across simulated distances and ship length conditions. The individual dots show the hit rates for each combination of ship length and distance that was simulated. Note the different *y*-limits in the left and right lower plots.

We now extend the principles of figure 8 to a simulation analysis using the details relevant to our experiment. Figure 9 shows simulation results for each camouflage type collapsed over ship length and range, and with the illustrative stochastic variability described above. The polar angle represents the ship’s direction of travel, and the distance from the origin represents the proportion of hits (the perimeter being 100% hit rate). Successful torpedo strikes fall within the bounded regions. Consistent with figure 8, the main areas of vulnerability are around east and west. Torpedoes aimed at ships travelling away from (between 300° and 60°) or towards (between 240° and 120°) the submarine are rarely successful. This is not surprising because the ship subtends a smaller visual angle under these conditions and so bias and variability in (simulated) perceptions will have greater impact on the hit rate.

The polar plot in figure 9*a* shows subtle differences in the positions of the east and west lobes across camouflage condition which are attributable to differences in *h* and *w*. Nonetheless, the overall accuracy of attacks varied little across camouflage condition (see the different coloured items in the lower whisker-plot). The regular circles and parti-colour conditions have the greatest and smallest value of *w,* respectively (figure 7), and these correspond with the lobes that are farthest apart in figure 9*a* (orange-dashed and green). We considered the influence of camouflage on the lobe positions further in figure 9*b* by comparing two conditions with opposing (positive and negative) *w* parameters. The positive twist (brown curves) draws the hit region further south, reflecting how interactions of twist and hysteresis combine beneficially or destructively at different points of the compass (refer also figures 6 and 8). This further supports our suggestion (refer §2) that the influence of twist depends on the direction of the camouflage texture gradient relative to the bow, though the overall effect (lower plot in figure 9*b*) is modest.

A distinct feature of the polar plots in figure 9 is the high hit rates for directions at or around 0° and 180°. This happens because the decay property of our descriptive model sets the direction errors to zero for these conditions (refer §3.2). Furthermore, variation in perceived speed is immaterial for head-on attacks so long as the ship is within the range of the torpedo. The limited range of the torpedo is why the simulation results fall shy of 100% for a direction of 0° in figure 9*a* and for the northerly part of the plot for the black curve in figure 9*b*, where the faster (longer) ships outrun the torpedoes. Since we found some differences across simulated conditions in this respect (i.e. different widths of the southerly spikes in figure 9) we chose to remove these regions (±5° from 0° and 180°) from our calculations for cumulative hit rates around the compass (the lower plots in figure 9).

In summary, the combination of twist and hysteresis measured in our experiments for patterned camouflage combine to deliver little or no overall benefit for ships’ crews compared to a treatment of neutral grey. This outcome owes to a complex interaction of factors, including the following: (i) hysteresis for camouflage patterns is less than for neutral grey (figure 7); (ii) hysteresis affords much of the protection in our simulations, the effects for twist being smaller (figures 7 and 8); and (iii) twist and hysteresis can combine both constructively and destructively which tends to nullify the potential benefit from twist (figure 6). However, following further consideration of expertise, we came to revise this conclusion as we discuss below.

#### 3.5.3. The influence of expertise, ship length and distance

Our analysis of the behavioural data revealed that hysteresis decreased with sailing experience (§3.3), although it did not have a significant effect on twist. This is consistent with experienced sailors becoming more adept at incorporating weak pictorial evidence for relief into their estimates. To examine the consequence of experience more closely, we repeated our torpedo aiming simulations with hysteresis ranging from *h* =~7.5° to *h* =~35°. This covered the range seen in our experiment and extended to lower levels to accommodate the potentially relevant experience of WWI submarine captains. We then considered hypothetical camouflages with (i) no twist (*w* = 0°) and (ii) the largest negative twist from the experiment (*w* = −9.8°). We set decay to the grand mean across all participants (*d* = 30.4°) and ran simulated attacks over distances of 500, 750 and 1000 m to investigate how accuracy varied as a function of ship length, distance and hysteresis. Our choice of *w* = 0° did not derive from our own measurement of twist in the neutral grey condition (where *w* = −3.6°) but an assumption about the expected level for non-dazzle painted ships in open seas. Note that while our experimental results suggest a reduction of hysteresis with the application of Mauretania dazzle and irregular circles, we have not included that curious feature here. Our analysis is intended to make the best possible case for dazzle camouflage in the context of misperception of direction.

**Figure 10.**
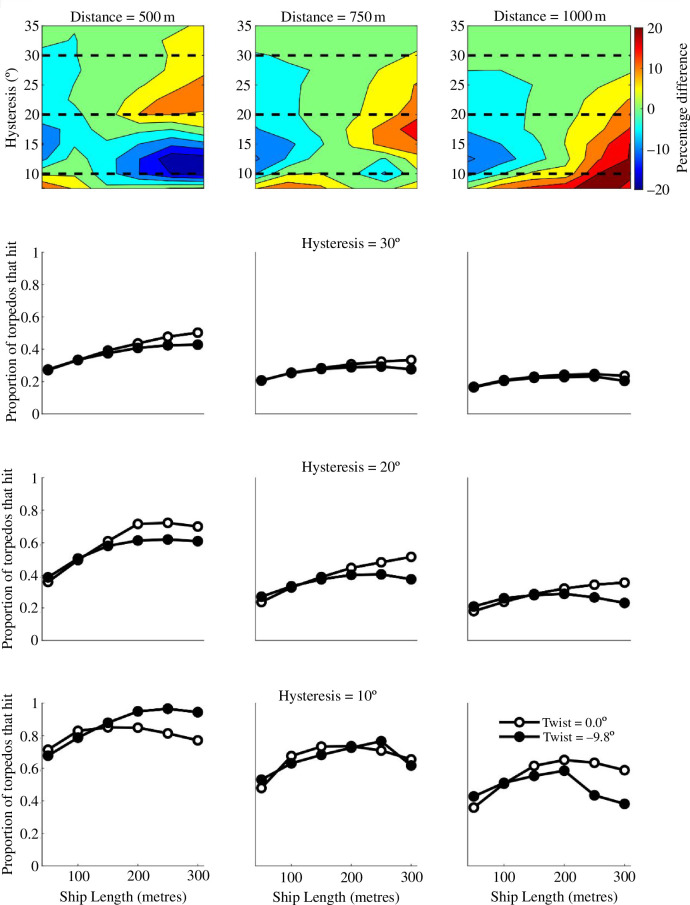
Simulated hit rates (averaged across ship directions) for different levels of twist (symbols) as functions of ship length. Different columns are for different distances, and in the lower plots, different rows are for different levels of hysteresis. The heat maps (top row) show the benefit of twist for various levels of observer hysteresis where the dashed lines correspond with the hysteresis in the plots below. The heat is derived by subtracting the hit rate for *w* = −9.8° from the hit rate for *w* = 0°. Hysteresis has an inverse relation with sailing experience (refer §3.5.3) but extends the values measured in our experiment (around 30°, refer figure 7) to as low as 10° here, in deference to the hypothetical experience of WWI submariners. The two values of twist are consistent with a perceptually strong dazzle texture gradient, as measured in our experiment (*w* = −9.8°), and a hypothetical camouflage with no twist (*w* = 0°).

The results are shown in figure 10. Unsurprisingly, there are more hits for shorter distances since perceptual errors are less important. More importantly, for longer (and therefore faster) ships, twist typically affords protection (filled symbols are lower than open symbols). This is particularly so for lower values of hysteresis (greater experience) at greater distances. The main exception is the combination of low hysteresis and near distance (lower left plot in figure 10) where twist is detrimental for the crews in longer ships, presumably owing to the cancellation with hysteresis as outlined in figure 8. The observations regarding distance, length and hysteresis are reinforced by the heat maps (top row of figure 10) where hotter colours denote greater protection.

One might suppose that because smaller ships make for smaller targets, this is where the errors would show. However, longer ships are also faster (refer §3.5.1) which means the targeting solutions inevitably involve more distant intersections, and this amplifies the consequence of errors in perceived direction.

This finding is consistent with a broader conclusion from [2] that: ‘[t]he [d]azzle system appeared to best suit large and fast vessels that operated alone or generally independent of the fleet or convoys’. It is difficult to obtain precise estimates of ship length from the theatre of WWI, but tramps varied in length between 76 and 121 m, were reportedly [27] the most frequently observed merchant ships in WWII, and date back to WWI, being one of the types investigated by Blodgett [8] in 1919. Troop ships, such as RMS Mauretania and SS Leviathan (also dazzle treated), were much longer (241 and 290 m, respectively) and this is where the benefit of an appropriate dazzle scheme, as investigated here, would have been at its best so long as the targeting distance was not short. However, few (if any) WWI dazzle schemes had the powerful texture gradients of our regular circles design (figure 2), though these ideas became evident between the wars [2].

## 4. General discussion

Dazzle camouflage was introduced in WWI to protect allied shipping from submarine attacks. We investigated the potential benefits of dazzle here by concentrating on perceived direction. We found two main effects that sum linearly: (i) a systematic twist of perceived direction induced by texture gradient-like camouflages; and (ii) a hysteresis effect for all ship treatments, including neutral grey. We found that regardless of the type of camouflage, ships were harder to hit for directions outside of about ±25° relative to the horizon (refer figure 9). We also found that the potential benefit of twist (in terms of a bias to perception of heading) was typically neutralized by hysteresis, though hysteresis always diminished in the presence of the (dazzle) patterns we tested. Hysteresis also diminished with sailing experience, whereupon the effectiveness of twist grew, having the greatest effect for larger ships.

### 4.1. A comparison with other studies

There are few other studies on the effects of dazzle camouflage on shipping. As we mentioned in §1, Blodgett [8] conducted an elaborate study shortly after the war (at the Boston District camouflage section of the U.S. Navy), long before the benefits of computerized displays. He reported numerous cases in which the misperception of direction for camouflaged model ships was far greater than for neutral ships (~40–60° or more). However, unlike here, true direction was not manipulated systematically, and it is difficult to judge whether he was measuring twist or hysteresis. In general, it is difficult to make reliable interpretations of what Blodgett found based on his presentation (for a brief summary, refer [5], p. 285).

In a much more recent study, Bekers *et al.* [6] applied dazzle camouflage to three-dimensional computer models of ships under various simulated weather conditions and tested them against an object recognition algorithm, trained on uncamouflaged ships. They found that in bright weather conditions, the application of dazzle to two test ships caused the accuracy of the recognition algorithm to fall from 72% to 56% correct. Under poor weather conditions, the accuracies converged (though values were not reported). This goes some way to supporting the notion that, twist and hysteresis aside, dazzle could benefit ship’s crews by interfering with class recognition and therefore estimates of range and speed.

### 4.2. Historical assessment of WWI dazzle and the implications of our work

While published experiments and statistics on the benefits of dazzle are close to non-existent (refer §4.1), classified research was conducted by the British and the USA during 1918–1919. Loyd A. Jones of the Eastman Kodak Company, Rochester, one of the US dazzle facilities, concluded that distortion of perspective was the only matter of value [2]. Wilkinson made a related point, claiming that ‘[t]he accurate estimation of a vessel’s course is the prime factor …’ and that ‘In every dazzle design this point was studied to the exclusion of all others, i.e. to frustrate accurate calculation of course’ [28]. From published images [1,2,5], it seems the Americans [5] became far more adept in the application of this principle than the British.

Despite the naval ambitions above, our work suggests that many WWI dazzle patterns would have been of limited benefit in systematically deceiving the enemy about a target ship’s direction. Indeed, our application of Wilkinson’s pattern for RMS Mauretania produced only 1° more twist than the neutral grey condition. The best camouflage, in terms of twist, was the regular circles design which was derived from the direct application of forced perspective (the texture gradient). Reportedly, several US designs used this principle [2] and these could have been effective.

After the 1918 report by the Committee of Enquiry on Dazzle Painting, the British Admiralty concluded that ‘… dazzle painting cannot possibly assist the submarine …’ [2]. Our results suggest this might not have been the case because high contrast patterns can (i) interfere with the protective benefits of hysteresis by distorting (twisting) the ships perceived heading and (ii) perhaps also decrease the magnitude of hysteresis. On the other hand, we also found that hysteresis diminishes with sailing experience. In sum, while we cannot know how WWI submariners would compare to even our most experienced participants, we speculate that for the high-stakes submariners, their hysteresis effect could have been lower, making the twist effect potentially more valuable for ships' crews at long distances (figure 10, bottom right).

Our work here was designed to investigate the effects of perceptual bias. However, an important component that we have not addressed, but was recognized in a 1919 report by Lieutenant Harold van Buskirk [2], is that dazzle schemes might also lead to confusion about direction without imposing a systematic bias. In principle, ambiguity might have been the motivating factor behind some of Wilkinson’s designs and those of others [5]. Finally, our torpedo modelling was based on displacement hulls where speed is inextricably linked to ship length. Many modern smaller vessels have a planing hull where speeds are not limited in this way. Such vessels would presumably reap greater benefit from dazzle camouflage, albeit, only in the context of WWI tracking technology or visually sighted weapons such as deck guns.

## 5. Conclusions

We have presented a systematic psychophysical enquiry into the effectiveness of dazzle camouflage on ships. We found both benefits and problems associated with the dazzle approach, particularly due to the interactive effects of hysteresis and twist. Nonetheless, we conclude that if fast ships were painted with camouflage that introduced a strong twist, it might have introduced targeting errors for experienced submariners which could have saved lives.

## Data Availability

All data and analyses are shared upon the Open Science Framework [29]. Supplementary material is available online [30].

## Footnotes

^1^
The term dazzle pre-dates its supposed widespread introduction in 1917. Taylor [1] points to evidence for its use in 1915. See also electronic supplementary material, S3.

^2^
The numbers on allied hits from U-Boats come from uboat.net (https://uboat.net/wwi/ships_hit/losses_year.html).

^3^
We note that some of the irregular circles appear to have an extended region of touch which we attribute to minor subpixel artefacts in the rendering.

^4^
Hull speed (*S*) in knots is given by: *S* = 1.35(√(3.28 L)) where *L* is the length of the ship in meters. Hull speed (*H*) in kph is given by: *H* = 1.852 *S*. In our simulations, we set ship speed to be 75% of hull speed.
